# Antiangiogenic effects in patients with progressive desmoplastic small round cell tumor: data from the French national registry dedicated to the use of off-labeled targeted therapy in sarcoma (OUTC’s)

**DOI:** 10.1186/s13569-017-0076-4

**Published:** 2017-05-10

**Authors:** Sarah Bétrian, Christophe Bergeron, Jean-Yves Blay, Emmanuelle Bompas, Philippe A. Cassier, Laure Chevallier, Jérome Fayette, Magali Girodet, Cécile Guillemet, Axel Le Cesne, Perrine Marec-Berard, Isabelle Ray-Coquard, Christine Chevreau

**Affiliations:** 1 Departments of Oncology and Clinical Research, Institut Claudius Regaud, Institut Universitaire du Cancer, Toulouse Oncopole, 1 avenue Irène Joliot-Curie, 31059 Toulouse Cedex, France; 2 Departments of Oncology and Clinical Research, Centre Léon Berard and Institut d’Hématologie et d’Oncologie Pédiatrique, Lyon, France; 3 Departments of Oncology and Clinical Research, Centre Léon Berard, Lyon, France; 4 Departments of Oncology and Clinical Research, Institut de Cancérologie de l’Ouest, Rene Gauducheau, St Herblain, France; 5 Clinical Research Department, CRA, Centre Léon Berard, Lyon, France; 6 Departments of Oncology and Clinical Research, Centre Henri Becquerel, Rouen, France; 7 Departments of Oncology and Clinical Research, Institut Gustave Roussy, Paris, France

**Keywords:** Desmoplastic small round cell tumor, Antiangiogenic, Chemotherapy treatment, Sunitinib

## Abstract

**Background:**

Desmoplastic small round cell tumor (DSRCT) is a very rare mesenchymal tumor that mainly affects teenagers and young adults with a mean age at diagnosis around 20–25 years. Although initial management still needs standardization, many centers will use multimodal treatment including intensive chemotherapy, extensive surgical resection followed by radiotherapy. Despite this, prognosis remains very poor and the median overall survival is 25 months. Recurrent disease is mainly treated by chemotherapy. Recently, due to the unmet medical need for recurrent disease, targeted therapies were explored for DSRCT.

**Methods:**

In this study, we assessed the response rate and progression free survival in nine cases of progressive DSRCT included in the OUTC’s registry and treated with antiangiogenics targeted agents (sunitinib, sorafenib and bevacizumab). OUTC’s, a French national registry, collects data about the use of off-label targeted therapy in sarcoma.

**Results:**

Eight males and one woman were included, with median age at diagnosis of 27.3 years (range from 9 to 48 years). They received a mean 3 lines (2–5) of treatment before antiangiogenic agent initiation. Six patients received sunitinib, two received sorafenib and one bevacizumab. Median progression free survival was 3.1 months (range 2–5.5 months) and best response observed was 5.5 months stable disease. Most patients had manageable low-grade toxicities, mainly fatigue, abdominal pain and skin toxicity.

**Conclusions:**

Despite very limited activity of antiangiogenics in our study, prospective collection of cases of these rare tumors together with molecular data should guide therapeutic decision and enhance outcome.

**Electronic supplementary material:**

The online version of this article (doi:10.1186/s13569-017-0076-4) contains supplementary material, which is available to authorized users.

## Background

Desmoplastic small round cell tumor (DSRCT) was first described in 1989 by Gerald and Rosai [1], as a tumor made of small round blue cells separated by abundant desmoplastic stroma. Fewer than 800 cases have been reported in the literature. DSRCT is therefore rare. DSRCT is an aggressive tumor, which mainly affects teenagers and young adults, predominantly males (sex ratio 4:1 to 9:1) with a peak incidence in the third decade of life.

On histological examination, tumor cells may display epithelial, mesenchymal and neuronal markers [2]. DSRCT is associated with a unique translocation t(11:22) (p13, q12) resulting in a fusion of the EWSR1 and the Wilm’s tumor WT1 genes. The chimeric product acts as an aberrant transcriptional factor and several transcriptional oncogenic targets have been identified including platelet derived growth factor A (PDGFA), insulin like growth factor 1 (IGF1) and WT-1 [3]. However, this has not yet been translated in efficient targeted therapy in this disease.

DRSCT typically involves the abdominal and peritoneal cavity, but extra-peritoneal organs may be affected [4–6]. Retroperitoneal lymph nodes, liver or lung metastases occur up to 50% cases at diagnosis [7]. There is no validated disease staging system, although the peritoneal cancer index (PCI) may be used for peritoneal disease [8] and Hayes-Jordan et al. have proposed a staging system specific to DSRCT [9].

With only one large case series described, the best therapeutic strategy remains unclear. DSRCT has a poor prognosis with a median OS of 25 months [7] and a 29 and 18% 3 and 5-years survival rate [10, 11]. A few small series suggest that intensive chemotherapy followed by extensive debulking surgery and abdominal radiation may improve the outcome of patients with operable disease [7, 12, 13]. Polychemotherapy schedule using alkylating agents and anthracyclins such as P6 protocol (cyclophosphamide, doxorubicin, vincristine, ifosfamide, etoposide, irinotecan and platinum alternating schedule) is associated with better outcome. A few centers have advocated the use of hyperthermic intraperitoneal chemotherapy (HIPEC) but, in the absence of randomized study, it is difficult to decipher the actual treatment effect from a selection bias in these series [14, 15]. Whole abdominal pelvic radiation therapy (WAP RT) and increasing development of intensity modulated radiation therapy were also described as a part of the adjuvant treatment [16, 17].

Overall, despite multimodal strategy, long term survivor rate remains low, with fewer than 20% of patients achieving 5-year survival [12]. Tumor recurrence and progression is the rule. After first line treatment, several cytotoxic agents have been studied, alone or in combination, including vinorelbine and cyclophosphamide [18], irinotecan [19], trabectedin [20, 21]. However, response rates are low and only partial responses are described and response duration remains short, range from 3 to 9 months.

Recently, there has been interest in the potential role of antiangiogenic tyrosine kinase inhibitors. Indeed, expression of EWS-WT1 induces PDGFA, a potent mitogen for fibroblast and endothelial cells. Particularly, PDGFA blockade may be interesting in DSRCT because of a profuse stromal reaction and neoangiogenesis surrounding tumor cells [3]. Thus, tyrosine kinase PDGFA receptor (PDGFRA) inhibitors such as imatinib and multikinase inhibitors, sunitinib and pazopanib, have been tested in DSRCT [22–24]. Our study reports nine cases of progressive DSRCT treated with antiangiogenic targeted agents, and registered in the national registry “OUTC’s” dedicated to the use of off-labeled targeted therapy in sarcoma.

## Patients and methods

### Registry

OUTC’s registry (Observatoire de l’Utilisation des Thérapies Ciblées dans le Sarcome) aims to collect, in a prospective manner, all medical data regarding the use of off-label targeted therapies in sarcoma to assess their efficacy and toxicity profile [25]. Off-label prescription is authorized in France for rare disease under control of experts, based on published data reporting potential activity.

### Patients

Patients with confirmed progressive DSRCT, not amendable to curative treatment, were included. As our study was not interventional, written consent was not required. However, patients were informed and gave oral consent for data collection and use of clinical data for research purposes. All patients received a detailed information letter and had the opportunity to withdraw their consent at any time. Children could be included with their parent’s consent. Progressive DSRCT was confirmed, in seven of our nine cases, by a systematic pathological review as part of two databases dedicated to collection and management of cases of soft tissue and visceral sarcomas (REePS: https://rreps.sarcomabcb.org/; and European Conticabase: https://conticabase.sarcomabcb.org/). All data were collected by the coordination center (Centre Léon Bérard, Lyon) after approval of the Centre Léon Bérard Clinical Trial Review Committee and two French data protection authorities (CCTIRS: Additional file 1; and CNIL: Additional file 2). Five university hospitals (all members of the GSF-GETO) participated and included all consecutive patients. Once a patient was registered, follow-up was established every 2 months by the coordination center.

### Study endpoints

The primary objective was to describe the efficacy of antiangiogenics agents in advanced DSRCT. Efficacy endpoints included response rate [i.e. partial and complete response (PR, CR)], according to RECIST criteria, disease control rate (i.e. rate of CR, PR and stable disease (SD) as best overall response) and progression free survival (PFS) under treatment. Secondary objective included characterization of safety profile.

### Statistical methods

Descriptive analysis was done using median values and range. PFS was defined as the time from start of AAT until progressive disease according to RECIST, or death. Safety evaluation was based on the frequency and severity of toxicities, graded according to the common terminology criteria for adverse events.

## Results

### Patient characteristics

From July 2002 to July 2013, 9 patients in 5 institutions were registered and received antiangiogenics targeted therapies (AAT) for advanced desmoplastic tumors. Patient and tumor characteristics, tumor staging and treatment are summarized in Table 1. There were 8 males. The median age at diagnosis was 27.3 years (range 9–48 years) and two patients were under the age of 18 years at diagnosis. Most patients had metastatic disease at diagnosis and have received multimodal treatment. Seven patients had surgical debulking of the primary tumor. First-line chemotherapy was generally based on anthracycline, ifosfamide and methotrexate. Three patients received radiotherapy as part of first line treatment. Median PFS after first line treatment was 19.5 months (range 6–47 months). Relapsed and progressive disease was treated with various chemotherapy regimens containing platinum, gemcitabine, irinotecan, temozolomide or trabectedine. Overall, for relapsed disease, complete response was observed for 2 first relapsed patients, with HIPEC and FOLFIRI regimen for one and etoposide, carboplatine, thiotepa and busulfan for the other one (PFS 17 and 12 months respectively). For the other patients short disease stabilization was the best response obtained with these second line regimens. Overall, second line treatment was associated with a median PFS of 10.5 months (range 2–17 months).

**Table 1:** Patient and disease characteristics

	Total (n = 9)
Sex	
Male	8
Female	1
Age at histological diagnosis (years)	
Mean (SD)	27,3
Range (years)	9–48
Tumor localization	
Abdomen	7
Head and neck	1
Pelvis	1
Histological subtype	
DSRCT	4
Desmoplastic medulloblastoma	1
Desmoplastic tumor with multiple differentiation	2
Unclassified desmoplastic tumor	2
Tumor stage (AJCC/IUCC)	
Unknown	2
IV_B_	7
Metastases at diagnosis	
Yes	6
Liver	4
Peritoneum	2
Other	1
Surgery of primary tumor	
Yes	7
Resection quality	
R_0_	0
R_1_	3
R_2_	2
Unknown	2

*SD* standard deviation, *AJCC* American Joint Committee on Cancer, *IUCC* Union for International Cancer Control

The median time from diagnosis to AAT was 3.6 years (range 10–78 months). Two patients received others targeted therapies, in 3rd line treatment, as part of a clinical trial (Ridaforolimus, mTOR inhibitor/SUCCEED trial *NCT00538239*, Dalotuzumab, IGFR1 inhibitor/DALORI trial) before they both received sunitinib. The median number of previous chemotherapy treatments before AAT was 3 (range 2–5). All patients had confirmed progressive disease at the time of initiation of AAT. In seven cases the decision to use off-label AAT was made after discussion in a multidisciplinary meeting. Six patients received sunitinib, two patients received sorafenib and one received bevacizumab. All treatments are summarized in Table 2.

**Table 2:** Treatment response and duration

Patient	First treatment	Surgery resection quality	Radiotherapy	PFS 1	Relapses treatments/*PFS*	Lines of treatment before TT	TT type	PFS with TT (months)
1	MAID^a^-ASCT^b^	R2	_	12 months	Gemcitabine-cisplatine/2,5 months	2	Sunitinib	2
2	LV5Fu-Ciplatine	_	_	8 months	HIPEC^c^-FOLFIRI^d^/17 months FOLFIRI/3 months Holoxan-etoposide/3 months Adriamycine-cyclophosphamide/*2* *months*	*5*	Sorafenib	3.5
3	MAI^e^	R1	Yes	39 months	Gemcitabine-docetaxel/4 months Adriamycine-holoxan/3 months Cisplatine-irinotecan/6 months Trabectedine/9 months	5	Sorafenib	4
4	Adriamycine-etposide-Cisplatine-Cyclophosphamide + VAI^f^	R2	_	44 months	Carboplatine-etoposide/13 months	2	Sunitinib	5.5
5	Cyclophosphamide-etoposide-carboplatine	R1	Yes	47 months	Etoposide-carboplatine-busulfan-thiotepa/12 months Temozolamide/5 months TEMIRI^g^/*4* *months*	4	Bevacizumab	2
6	MAI	_	_	6 months	VAC^h^/6 months	2	Ridaforolimus *PFS *23 months Sunitinib	4 OS 38 months
7^j^	Bevacizumab-IVADo^i^	R1	Yes	7 months	Navelbine-cyclophosphamide/10 months	2	Dalotuzumab *PFS *2 months Sunitinib	3
8	Adriamycine-ifosfamide-etoposide	_	_	6 months	Cyclophosphamide/3 months Trabectedine/4 months	3	Sunitinib	2 OS 19 months
9	MAI	_	_	7 months	VAC/3 months	2	Sunitinib	2

^a^Mesna-Adriblastine-Ifosfamide-Dacarbazine

^b^Autologous stem cell transplantation

^c^Hyperthermic intraperitoneal chemotherapy

^d^5 Fluorouracile-Oxaliplatine-Irinotecan

^e^Mesna-Adriblastine-Ifosfamide

^f^Vincristine-Actinomycine D-Ifosfamide

^g^Temozolamide-Irinotecan

^h^Vincristine-Actinomycine D-Cyclophosphamide

^i^Ifosfamide-Vincristine-Actinomycine D-Doxorubicine

^j^This patient had TT bevacizumab in first line treatment as part of BERNIE study in combination with IVADo polychemotherapy. The second relapse was treated with dalotuzumab, he received temodal-irinotecan for third relapse (PFS 8 months) and he finally had sunitinib for the fourth relapse

### AAT efficacy

Efficacy data were collected every 2 months. Individual responses to previous treatments and AAT are detailed in Table 2. The median PFS was 3.1 months for patients who received sunitinib. Overall, for the nine patients treated with AAT, the disease control rate at 2 months was 66.7% and PFS was also 3.1 months. The patient treated with bevacizumab died from progressive disease at 2 months and the patients treated with sorafenib had progressive disease at 4 months. Overall, for these nine patients, the best response had been stable disease.

### AAT safety

The main toxicities are presented in Table 3. Among the six patients treated with sunitinib, gastro-intestinal toxicity and fatigue were the most common adverse events (AEs) (n = 4). One patient had grade 3 hepatitis, but otherwise, most AEs were low grade and none required interruption of sunitinib. One patient treated with sorafenib had grade 1 skin toxicity and grade 2 cough. The other patient treated with sorafenib had grade 3 abdominal pain that required cessation of treatment. Overall, the toxicity profile seen with these agents was similar to that observed in others studies [26]. There were no grade IV adverse events and no toxic deaths occurred during follow-up.

**Table 3:** TT toxicities

Sunitinib (n = 6)	Total N	Grade 1 N	Grade 2 N	Grade 3 N
Skin toxicity	1	1	0	0
Fatigue	3	2	0	1
Nausea	1	1	0	0
Diarrhea	1	1	0	0
Abdominal pain	1	1	0	0
Hepatic cytolysis	1	0	0	1
Dyspnoea	1	0	0	1
Hematoxicity	2	*1* (*anemia*)	0	1 (*lymphopenia*)
Sorafenib (n = 2)				
Skin toxicity	1	1	0	0
Abdominal pain	1	0	0	1
Cough	1	0	1	0

## Discussion

Relapsed and refractory DSRCT remains associated with very poor prognosis. As noted above, multimodal therapy that combines polychemotherapy, debulking surgery and radiotherapy is associated with better outcome than less comprehensive treatment [7, 12].

In our case series, clinical patients characteristics are comparable to those of previous series. Seven of our nine patients had debulking surgery even in presence of metastatic disease at diagnosis. First-line chemotherapy was based on anthracyclines, ifosfamide and etoposide using regimens adapted from the P6 protocol. These approaches are similar than those used in the most studies reported to date and are considered as standard care. The median PFS following first-line treatment was 19.5 months, which is comparable with PFS reported in the literature [11, 27].

There is no standard approach beyond first line, and patients received in most cases chemotherapy and salvage surgery when feasible. Gemcitabine, temozolamide, irinotecan and trabectedine were the main cytotoxic agents used. Overall, second line treatment was associated with a median PFS of 10.5 months (range 2–17 months). The only one patient who was treated with sunitinib monotherapy had a PFS of only 2 months. These data suggest that, if performance status allows, treatment for relapsed disease should again be based on combination chemotherapy.

Recent data suggest the interest of novel targeted therapy, against several biological processes involved in DSRCT pathogenesis, to treat recurrent diseases. Based on Ewing sarcoma model and EWS-Fli translocation, leading to dysregulation of insulin growth factor 1 (IGF1) receptor and dependence on IGF1 [28, 29], humanized monoclonal antagonist IGF1 receptor (IGF1R) antibodies have been developed [30, 31]. As part of clinical trial, one of our patients received the IGF1R antibody dalotuzumab, with a low PFS.

The modest efficacy of these signaling inhibitors and the development of resistance provide a rationale for the use of antiangiogenic multi-targeted kinase inhibitors. Only a few AATs have been tested in patients with relapsed and refractory DSRCT. Imatinib was used in a phase II study for two patients with DSRCT expressing KIT and/or PDGFRα with very limited response [22]. This may be explained by the fact that KIT expression probably leads to tumorigenicity as a spectrum, with more reliance on other kinase pathways, and may be targeted by other tyrosine kinase small molecules inhibitors [22]. Sunitinib is a multi-targeted agent that potently inhibits PDGFR, KIT, FLT3, CSF-1 and RET, as well as vascular endothelial growth factor (VEGF) receptor tyrosine kinases. It targets multiple signaling pathways in tumor, stromal, and endothelial compartments that are relevant to DSRCT [32]. Six of our patients received sunitinib as AAT for progressive disease with a median PFS of 3.1 months. Thus, sunitinib activity seems to be comparable to recent eight cases reported by Italiano et al. with median PFS of 2.6 months [23].

Overall, our results are similar to those described in the other retrospective analyses of monochemotherapy and most AAT for progressive DSRCT (Table 4). Efficacy was modest and the best response obtained was stable disease. However, a retrospective study of pazopanib in nine DSRCT cases reported promising results with a 9.2 months PFS [24]. Such as sunitinib, pazopanib is an orally available inhibitor of the tyrosine kinases of several factors including the vascular endothelial growth factor receptors (VEGFR) 1–3, c-KIT, and the platelet-derived growth factor receptors (PDGFR) alpha and beta, and has been approved for advanced soft tissue sarcoma.

**Table 4:** Literature data of chemotherapy agents and TT for advanced TT

Chemotherapy agent	Patients number	Best response	PFS	References
Vinorelbine-Cyclophosphamide	2	2 PR	4 and 15 months	Ferrari et al. [18]
Irinotecan	8	4 SD, 4 PD	Unknown	Bisogno et al. [19]
Trabectedine	2	2 SD	4 months	Frezza et al. [21]
TT agent				
Imatinib	7	1 PR, 4 PD, 2 NA	1 month	Chao et al. [22]
Sunitinib	8	2 PR, 3 SD, 3 PD	2.6 months	Italiano et al. [23]
Pazopanib^c^	9	2 PR, 5 SD, 2 PD, CBR^a^ 78%	9.2 months	Frezza et al. [24]
Temsirolimus	1	SD	10 months	Thijs et al. [33]
Ganitumab	16	1 PR, 10 SD, 4 PD, CBR^b^ 25%	19 months	Tap et al. [30]

*PR* partial response, *SD* stable disease, *PD* progressive disease, *NA* non assessable, *CBR* clinical benefit rate (PR + SD >12 weeks^a^, 24 weeks^b^)

^c^Retrospective analysis from data comprising three DSRCT patients treated within the EORTC phase II study 62043, three in the EORTC phase III study 62072 (PALETTE), along with three patients treated in the UK on the subsequent pazopanib named patient program

This cohort, although small and retrospective, confirms the safety of AATs. AEs were mostly low grade and manageable, similar to those previously reported in other series.

Moreover, recent data on olaratumab, a recombinant human IgG1 monoclonal antibody that specifically binds PDGFRA and receptor activation, and its FDA approval, may significantly improve outcome of patients with soft tissue sarcoma. In this study, only one patient had an undifferentiated round cell sarcoma negative for EWS in olaratumab treatment arm [34].

## Conclusions

OUTC’S program allows the collection of data on the off-label use of TTs in rare tumors such as DSRCT. This is especially useful since such patients are generally ineligible for clinical trials. Moreover new treatment development in DSRCT remains poor. Despite negative results, this study suggests that AAT with sunitinb, sorafenib or bevacizumab monotherapy doesn’t seem to be the best approach to treat progressive DSRCT. Identification of molecular pathways and specific mutations involved in DSRCT pathogenesis may allow the development of new targeted and combination treatments to improve response rate and survival in relapsed and progressive DSRCT. Thus, IGFR1 inhibitors and multi-targeted kinase inhibitors may be another treatment strategy, probably in combination with chemotherapy. Prospective collection of cases of these rare tumors treated with targeted therapies together with increased molecular data understanding should improve therapeutic decision and enhance outcome.

## Additional files

**Additional file 1.** CCTIRS (Comité Consultatif sur le traitement de l’information en matière de recherché dans le domaine de la santé) approved the relevance of the personal name specific data with regard to the objective of the research.

**Additional file 2.** CNIL (Comité National d’Information et Libertés) French committee in charge of watching that the computing is in the service of the citizen and that it strikes a blow neither at the human identity, nor at the human rights, nor at the private life, nor at the personal or public freedoms.

## Abbreviations

DSRCT: desmoplastic small round cell tumor
PDGFA: platelet derived growth factor A
PDGFRA: platelet derived growth factor receptor A
IGF1: insulin like growth factor 1
WAP RT: whole abdominal pelvic radiation therapy
HIPEC: hyperthermic intraperitoneal chemotherapy
AAT: anti-angiogenic targeted therapy
