# Supplementary material for: Antiangiogenic effects in patients with progressive desmoplastic small round cell tumor: data from the French national registry dedicated to the use of off-labeled targeted therapy in sarcoma (OUTC’s)
Source: Clin Sarcoma Res. 2017 May 10;7:10. doi: 10.1186/s13569-017-0076-4 (PMC5424317; doi:10.1186/s13569-017-0076-4)
Supplement: Supplementary file 1 — Additional file 1. CCTIRS (Comité Consultatif sur le traitement de l’information en matière de recherché dans le domaine de la santé) approved the relevance of the personal name specific data with regard to the objective of the research. [file 13569_2017_76_MOESM1_ESM.pdf]

Liberté • Égalité • Fraternité
RÉPUBLIQUE FRANÇAISE

ministère
enseignement
supérieur
recherche

DIRECTION GENERALE DE LA RECHERCHE
ET DE L'INNOVATION

Comité consultatif sur le traitement de l'information en matière de recherche dans le domaine de la santé

Dossier n° : 09.095bis

Intitulé de la demande : Observatoire de l'Utilisation des Thérapeutiques Ciblées dans le domaine des Sarcomes.

Demandeur : Centre Léon Bérard
Monsieur Thierry PHILIP
28, rue Laënnec
69373 LYON Cedex 08

Responsable : **Isabelle RAY-COQUARD**

Dossier reçu le : 6 mai 2009

Dossier examiné le : 4 juin 2009

Avis du Comité consultatif :

Avis favorable

Fait à Paris, le 11 juin 2009

Le Président du Comité consultatif

Mahmoud ZUREIK

1, rue Descartes – 75231 Paris Cedex 05
http://www.recherche.gouv.fr
