# Supplementary material for: Antiangiogenic effects in patients with progressive desmoplastic small round cell tumor: data from the French national registry dedicated to the use of off-labeled targeted therapy in sarcoma (OUTC’s)
Source: Clin Sarcoma Res. 2017 May 10;7:10. doi: 10.1186/s13569-017-0076-4 (PMC5424317; doi:10.1186/s13569-017-0076-4)
Supplement: Supplementary file 2 — Additional file 2. CNIL (Comité National d’Information et Libertés) French committee in charge of watching that the computing is in the service of the citizen and that it strikes a blow neither at the human identity, nor at the human rights, nor at the private life, nor at the personal or public freedoms. [file 13569_2017_76_MOESM2_ESM.pdf]

CNIL

Le Président

Madame Isabelle RAY-COQUARD
CENTRE LEON BERARD
CLB
EVALUATION DES PRATIQUES MEDICALES
28 RUE LAENNEC
69373 - LYON CEDEX 03

Paris. le - 2 DEC. 2009

N/Réf. : AT/YPA/SV/SN/GDP/AR091413

Objet : NOTIFICATION D'AUTORISATION

DEMANDE D'AUTORISATION N° 1375805
A rappeler dans toute correspondance

Madame,

Vous avez saisi le 17 juillet 2009 notre Commission d'une demande d'autorisation relative à un traitement de données à caractère personnel ayant pour finalité

OBSERVATOIRE DE L'UTILISATION DES THERAPEUTIQUES CIBLEES DANS LE DOMAINE DES SARCOMES

Ce traitement relève de la procédure des articles 54 et suivants de la loi du 6 janvier 1978 modifiée.

En conséquence, conformément aux dispositions de l'article 15 de la loi du 6 janvier 1978 modifiée relative à l'informatique, aux fichiers et aux libertés et après réception de vos compléments, j'autorise la mise en œuvre de ce traitement.

Je vous prie, Madame, d'agréer l'expression de mes salutations distinguées.

Alex TÜRK
